# Dermatology patient-derived health utility of facial angiofibroma associated with tuberous sclerosis complex

**DOI:** 10.1007/s11136-026-04362-1

**Published:** 2026-07-30

**Authors:** Jessie T. Lu, David K. E. Chan, Grace X. Li, Sophy T. F. Shih, Deshan F. Sebaratnam

**Affiliations:** 1 School of Clinical Medicine, University of New South Wales Medicine and Health, Sydney, NSW Australia; 2 Department of Dermatology, Liverpool Hospital, Liverpool, NSW Australia; 3 Mark Wainwright Analytical Centre, Stats Central, University of New South Wales, Sydney, NSW Australia

## Abstract

**Purpose:**

The majority of patients with Tuberous Sclerosis Complex have cutaneous manifestations, the most common being facial angiofibroma (FA). The impact of facial angiofibroma on quality of life has not been comprehensively evaluated, and a health state utility has not previously been determined. The purpose of this study was to calculate the health utility of facial angiofibroma from the perspective of dermatology patients and explore any associations between participants’ sociodemographic characteristics and the perceived utility of facial angiofibroma.

**Methods:**

In a structured interview format, participants recruited from dermatology clinics (*n* = 100) were asked to imagine themselves in each of four health states (monocular blindness, binocular blindness, almost-clear facial angiofibroma and moderate facial angiofibroma). Visual analogue scale, time trade-off and standard gamble were conducted for each health state and each outcome was converted into a health state utility.

**Results:**

The adjusted mean health state utilities were 0.88 [0.84,0.92], 0.95 [0.91,0.99] and 0.94 [0.91,0.98] for almost-clear facial angiofibroma, by Visual Analogue Scale, Time Trade-Off and Standard Gamble, respectively, and 0.68 [0.64,0.72], 0.88 [0.84,0.92] and 0.91 [0.87,0.94] for moderate facial angiofibroma. Participants theoretically elected to sacrifice 6.8 years of their remaining 57 years of life (12%) and accept a 9% risk of immediate death to receive curative treatment for moderate facial angiofibroma.

**Conclusion:**

The health state utility of moderate facial angiofibroma was significantly poorer than almost-clear facial angiofibroma. This highlights that reducing the severity of facial angiofibroma can deliver potential gains in quality of life.

**Supplementary Information:**

The online version contains supplementary material available at https://doi.org/10.1007/s11136-026-04362-1.

## Introduction 

Tuberous Sclerosis Complex (TSC) is estimated to affect up to 1 million people worldwide [1]. It is an autosomal dominant disorder caused by variants in the tumour suppressor genes, *TSC1* and *TSC2*, which code for hamartin and tuberin, respectively. These proteins form an intracellular complex which regulates the activation of the mammalian target of rapamycin (mTOR) protein [2]. In TSC, pathogenic variants in either of these genes result in uncontrolled cell growth and proliferation, manifesting as the development of hamartomas in multiple organs, preferentially affecting the skin, nervous system and kidneys [1].

Skin manifestations affect the majority of patients with TSC and often prompt initial diagnosis [3]. The most common cutaneous stigmata of TSC are facial angiofibroma (FA) [4]; small, grouped, erythematous papules, characteristically affecting the central face [5]. These benign tumours are composed of fibrovascular tissue and the presence of ≥ 3 FA in adolescence is highly suggestive of TSC [3]. Although benign, FA may be associated with morbidity, due to cosmesis, bleeding, pain, and less commonly, infection and obstruction of vision or nasal passages [6].

Traditional management of FA has involved procedural intervention including excision, curettage, dermabrasion, or laser ablation [5, 7]. These treatments may be associated with significant cost, recovery and risks of scarring, hyperpigmentation and recurrence [4]. The introduction of topical mTOR inhibitors has revolutionised the treatment of FA since 2010 [8], with an improvement in lesions seen in over 75% of patients [9] and demonstrating a favourable safety profile [10, 11].

Improvements in quality of life (QoL) have been shown through the 36-Item Short Form Health Survey where 3 months of treatment with topical sirolimus generated significant improvements in vitality, social function and mental health [12]. The health state utility (HSU) is a ‘score’ attached to a particular health state on a cardinal scale where 0 represents death and 1 represents perfect health [13], quantifying QoL burden. HSUs can be determined using direct or indirect methods. Direct methods consist of an interviewer/s presenting health states to a participant to elicit a preference based on their perception of these health states. Indirect methods, however, utilise multi-attribute utility instruments which bypass the need for participant interviews each time a study is conducted. Responses are converted into utilities via ‘tariffs’ using an algorithm calibrated against preferences of the general population [14]. While several indirect elicitation methods are widely available, it is difficult to evaluate the burden of conditions, such as FA, which do not have a broad impact on multiple areas of QoL. For instance, the EQ-5D includes mobility and self-care as part of the five fundamental aspects of QoL [15], while the SF-6D encompasses physical functioning, role limitations and vitality [16], all of which are unlikely to be significantly impacted by a facial condition. Such tools would lack sensitivity in valuations of QoL in FA, making direct elicitation more suitable for the purposes of this study.

HSUs can be used to estimate quality-adjusted life years (QALYs) and further applied to cost-utility studies, which examine the inputs required to generate an increase in QALYs. When compared to a country’s willingness-to-pay threshold, these results can then guide resource allocation [17]. HSUs can be derived from multiple perspectives, a narrow patient-perspective or a broader general population perspective. It is known that there are often discrepancies between HSUs derived from varying perspectives, however there is ongoing debate as to the most appropriate population from which to elicit preferences. It is argued that patients with lived experience of a health state have a greater understanding of its true severity and impact on QoL. However, deriving HSUs from patients potentially overestimates outcomes due to accommodation, or the response shift effect, and limits comparability with other health states [18]. The societal perspective is valued in economic evaluations to reflect the preferences of a society in a healthcare system of limited resources, rather than individual patient decisions, aligning with reference case recommendations [13, 19].

The objective of this study was to determine the HSUs of FA in TSC from the perspective of dermatology patients using three direct method assessment tools: visual analogue scale (VAS), time trade-off (TTO) and standard gamble (SG). It also sought to investigate any sociodemographic factors which demonstrated an association with the HSU output.

## Participants and methods

Over a 4-month enrolment period, adult participants with sufficient English language proficiency and without TSC-associated FA were recruited from public and private dermatology clinics in Sydney, Australia. The recruitment of participants from a community-facing setting was intended to be a pragmatic approach to recruit a study population with diverse sociodemographic characteristics. In a face-to-face interview with a trained investigator, participants were shown an image of each of four health states: monocular blindness, binocular blindness, almost clear TSC-associated FA and moderate TSC-associated FA. Participants were asked to imagine themselves in each health state whereby VAS, TTO and SG were completed sequentially in a fixed order.

### Health states

Two health state images of FA (Online Resource 1) were selected based on the Investigator’s Global Assessment scoring created by Aitken et al. [6] which grades FA from 0 to 4 on the basis of size and colouration. These images represented grade 1, or almost-clear FA, and grade 3, or moderate FA. The grading of these images was reviewed by the principal investigator, an experienced clinician with expertise in TSC. The study design assumed participant evaluation would be based solely on the health state depicted, hence no formal standardisation of individual characteristics, such as apparent gender, was conducted. The health state images of monocular and binocular blindness acted as internal controls to assess participants’ understanding and baseline risk tolerance. These are standard comparator health states previously employed in other published health utility studies [20–23] and enable contextualisation of FA, an externally visible condition, against blindness health states with more prominent functional implications.

### Visual analogue scale (VAS)

VAS is a routinely used tool, both as a standalone and in combination with other methods. Participants were shown a vertical linear scale with 100 representing perfect health, and 0 representing a state equivalent to death. The value assigned to a health state was converted to an HSU using the formula:

$$ {\mathrm{HSU}} = \frac{{{\mathrm{VAS}}}}{{100}} $$

### Time trade-off (TTO)

The TTO method requires participants to trade off a length of time to cure a health state, introducing an economic component which is intrinsic to everyday decision-making [24]. Participants were asked to imagine they had 57 years of life remaining. This value was chosen based on the difference between the average life expectancy in Australia of 83 years and 26 years being the average age of patients with FA in large-scale randomised control trials and published database analyses [4, 6]. An iterative ping-pong method was employed to vary the years traded off between 0 and 57 years, until a point of indifference was found. For instance, participants were first asked whether they would sacrifice 1 year of their remaining 57 years, followed by whether they would sacrifice 56 years, alternating between high and low values. The TTO score was determined using the following formula:

$$ {\mathrm{HSU}} = 1 - \frac{{{\mathrm{number}}\;{\mathrm{of}}\:{\mathrm{years}}\:{\mathrm{traded}}\:{\mathrm{off}}}}{{57}} $$

### Standard gamble (SG)

SG required participants to weigh up the risk they were willing to take on to cure a particular health state, again, given that they had 57 years of life remaining. An iterative ping-pong method was utilised, and the risk of death was varied between 0% and 100%, until a point of indifference was found. This value was converted into an HSU through the formula:

$$ {\text{HSU = 1}} - p $$

(where *p* is the risk of death at the point of indifference)

### Statistical analysis

Statistical analyses were performed using R version 4.5.0 [25]. HSUs were normalised to a standardised scale ranging from 0 (death) to 1 (perfect health) and the mean and standard deviation of HSUs for each tool and health state were calculated. The Shapiro-Wilk test was used to assess for normality of HSUs by health state and tool and Levene’s test was used to assess for homogeneity of variance of HSUs from an assessment tool between the four health states.

Simple linear regression (SLR) was used to explore bivariate associations between sociodemographic variables and HSUs by assessment tool and health state (almost-clear FA and moderate FA), where the sociodemographic variable was set as the explanatory and the HSU set as the response variable. For each sociodemographic variable, the estimated regression coefficient(s) and associated 95% confidence interval(s) were extracted from the respective SLR model. Statistical significance was assessed using p-values extracted from the F-statistic.

Sociodemographic variables which demonstrated evidence of an association with HSUs were included in the following multivariable modelling. A multivariable linear mixed effects model (LMM) was employed with HSUs as the response and a participant-level random-effect to account for repeated measurements of participants. The covariates were any sociodemographic variables which demonstrated consistent evidence of a bivariate association in the SLR, along with health state, assessment tool and an interaction term between health state and assessment tool. The multivariable LMM assumptions were checked with residual plots, and variance inflation factors were calculated to identify collinear sociodemographic variables.

The fitted multivariable LMM’s analysis-of-variance table for covariates was used to assess for significant sociodemographic variables. The estimated marginal mean HSUs for each health state were determined under each assessment method and post-hoc mean differences were calculated with Bonferroni’s correction. The significance level was set to 0.05 (two-tailed) for statistical inference.

## Results

A total of 113 adult patients were invited to participate in this study, of which 4 declined to participate. Of the 109 participants who completed the interview, 9 responses were excluded as they either (a) reported the same utility across all health states or (b) reported inferior utility for monocular blindness compared to binocular blindness in at least one assessment tool. The 100 remaining responses were included for statistical analysis. The sociodemographic characteristics of the participants are summarised in Table 1. The median age of participants was 44 years, with a higher proportion of females (*n* = 58, 58.0%) than males (*n* = 42, 42.0%).

**Table 1:** Sociodemographic characteristics of study participants (*n* = 100)

Characteristic	Participants
n	100
Age, median [IQR]	44 [31.8, 58.3]
*Sex, n(%)*	
Female	58 (58.0)
Male	42 (42.0)
*Ethnic Group, n(%)*	
Asian	30 (30.0)
White	59 (59.0)
Hispanic/Latino	2 (2.0)
Mixed race/Biracial	3 (3.0)
Other	6 (6.0)
Household Income (AUD), n(%)*	
<$50,200	14 (14.0)
$50,200-$116,000	25 (25.0)
>$116,000	50 (50.0)
Decline to Answer	11 (11.0)
*Education, n(%)*	
Some High School	6 (6.0)
High School Diploma	13 (13.0)
Some University/College	13 (13.0)
Bachelor’s Degree or Higher Education Qualification	37 (37.0)
Postgraduate Qualification	30 (30.0)
Decline to Answer	1 (1.0)
*Relationship Status, n(%)*	
Committed Relationship	66 (66.0)
Divorced/Separated	8 (8.0)
Single	23 (23.0)
Widowed	3 (3.0)

*Derived from the Australian Bureau of Statistics low-, middle- and high-income groups [26]

The standardised HSUs on a 0 (death) to 1 (perfect health) scale are summarised in Table 2 by assessment tool. The mean HSUs for moderate FA were lower than almost-clear FA for each assessment tool. HSUs for moderate FA were higher than binocular blindness but broadly similar to monocular blindness.

**Table 2:** Health state utilities for each health state by assessment tool

Tool		Monocular blindness	Binocular blindness	Almost-clear facial angiofibroma	Moderate facial angiofibroma
VAS	mean ± sd	0.73 ± 0.16	0.42 ± 0.21	0.88 ± 0.16	0.68 ± 0.21
	median (IQR)	0.70 (0.60–0.81)	0.40 (0.25–0.60)	0.93 (0.85-1.00)	0.70 (0.50–0.85)
TTO	mean ± sd	0.89 ± 0.14	0.75 ± 0.23	0.95 ± 0.10	0.88 ± 0.12
	median (IQR)	0.91 (0.88–0.98)	0.82 (0.65–0.91)	0.98 (0.95-1.00)	0.91 (0.82–0.98)
SG	mean ± sd	0.90 ± 0.12	0.82 ± 0.17	0.94 ± 0.11	0.91 ± 0.11
	median (IQR)	0.95 (0.90–0.99)	0.90 (0.70–0.95)	0.99 (0.95-1.00)	0.95 (0.90–0.99)

VAS = Visual Analogue Scale, TTO = Time Trade-Off, SG = Standard Gamble, sd = standard deviation, IQR = interquartile range

Sparse categories within the sociodemographic variables of ethnic group, level of education and relationship status were collapsed due to the relatively small sample size and to enhance interpretability. Exploratory analyses demonstrated an association between level of education and TTO (*p* = 0.007) and SG (*p* < 0.001) HSUs for almost-clear FA, and SG HSUs (*p* = 0.026) for moderate FA as shown in Table S1 (Online Resource 2). Level of education was included as a covariate in subsequent multivariable modelling. Compared with White ethnicity, non-White ethnicity was a significant predictor for the VAS HSU (*p* = 0.030) for almost-clear FA only. SLR did not show any univariate associations between age, sex, household income or relationship status and HSUs for FA.

In the subsequent LMM, level of education was not a significant predictor of HSUs (ANOVA: F(2,96) = 1.455, *p* = 0.239), however health state (ANOVA: F(3,1078) = 28.259, *p* < 0.001) and assessment tool (ANOVA: F(2,1078) = 286.185, *p* < 0.001) were significant predictors as seen in Table S2 (Online Resource 2).

Post-hoc pairwise comparisons between health states found that HSUs for almost-clear FA were significantly higher than moderate FA (difference = 0.103, 95% CI = 0.073 to 0.133, *p* < 0.001), as shown in Table S4 (Online Resource 2). The mean difference in HSU between monocular blindness and moderate FA was 0.019, however this was not a significant result (95% CI = −0.011 to 0.049, *p* = 0.542). When compared to binocular blindness, however, moderate FA HSUs were significantly higher (difference = −0.161, 95% CI = −0.191 to −0.131, *p* < 0.001).

Post-hoc analysis of pairwise differences in HSUs across health states by assessment tool was also conducted as shown in Table S5 (Online Resource 2). VAS produced HSUs which were lower than TTO by 0.132 (95% CI = −0.159 to −0.106, *p* < 0.001) and lower than SG by 0.143 (95% CI = −0.169 to -0.116, *p* < 0.001). However, there was no significant difference between the HSUs determined using TTO and SG (95% CI = −0.037 to 0.016, *p* = 1.000).

Although Shapiro-Wilk and Levene’s tests showed departures from normality and homoscedasticity, visual analysis of residual-fitted plots shown in Figures S3-S4 (Online Resource 1) revealed no major violations of the assumptions of multivariable LMMs, and variance inflation factors did not identify any significant multicollinearity. The level of education-adjusted mean HSUs across health states and by assessment tool are summarised in Table 3.

**Table 3:** Estimated marginal means of health state utilities from linear mixed-effects model, adjusting for education as a covariate

Tool	Monocular blindness		Binocular blindness		Almost-clear facial angiofibroma		Moderate facial angiofibroma	
	Adjusted mean	95% CI	Adjusted mean	95% CI	Adjusted mean	95% CI	Adjusted mean	95% CI
VAS	0.728	[0.689, 0.767]	0.417	[0.378, 0.456]	0.883	[0.844, 0.921]	0.681	[0.642, 0.720]
TTO	0.892	[0.854, 0.931]	0.750	[0.711, 0.789]	0.948	[0.909, 0.987]	0.880	[0.841, 0.919]
SG	0.904	[0.865, 0.942]	0.815	[0.776, 0.854]	0.943	[0.905, 0.982]	0.905	[0.867, 0.944]

VAS = Visual Analogue Scale, TTO = Time Trade-Off, SG = Standard Gamble, CI = confidence interval

## Discussion

Existing literature highlights the inverse correlation between severity of FA [27] and QoL and the positive impact of treatment for FA on QoL [28]. Previous research has estimated HSUs for TSC [29], but FA have been largely overshadowed by the significant burden of other manifestations of the condition, such as epilepsy. Emerging treatments for FA, such as topical sirolimus, will require formal economic evaluation, for which the first stage involves estimating FA-specific HSUs. Thus, this study is the first to calculate the HSU of FA associated with TSC using three standardised direct method assessment tools. These were derived from patients of dermatology clinics unaffected by FA, representing a proxy of the general population to reflect the preferences of consumers in a healthcare system with inevitably limited resources.

Our study results demonstrate that moderate FA is perceived to have a significantly greater burden on QoL than almost-clear FA but comparable to monocular blindness and significantly less than binocular blindness. Participants theoretically elected to sacrifice 6.8 years of their remaining 57 years of life (12%) for a treatment to cure moderate FA and were willing to undergo a treatment despite a 9% risk of immediate death (TTO: 0.88; SG: 0.91). In comparison, participants perceived almost-clear FA as having a significantly lesser impact on QoL and were willing to sacrifice just 3 years of life (5%) and accept a 6% risk of death for treatment (TTO: 0.95; SG: 0.94). Although meaningful, these values are derived from hypothetical health states and shaped by participants’ cognitive processing of probability and risk, inherently shaped by hypothetical bias. Therefore, comparison between health states may provide more meaningful interpretation of such findings. When examined alongside general population-derived HSUs for other conditions with facial aesthetic implications, moderate FA appears to be comparable to living with a nasal deformity (TTO: 0.87; SG: 0.91) [30] and more burdensome than both male (TTO: 0.95; SG: 0.96) and female (TTO: 0.93; SG: 0.95) alopecia [21]. In comparison to cleft lip and/or palate, a facial condition with a more notable functional impact (TTO: 0.85; SG: 0.84) [31], moderate FA had more favourable HSUs. While informative, a lack of methodological standardisation between these studies necessitates cautious interpretation of direct comparisons. Participants in these studies were recruited through various settings such as shopping centres, online sites and laboratory volunteers, and the data collection format differed between online surveys and in-person interviews.

The significant differences in HSUs of moderate versus almost-clear FA across all assessment tools suggests that treatments to reduce the severity of FA have the potential to deliver a health benefit in QoL. In a patient who lives for 57 years with FA, treatment to lessen the severity of lesions would represent a gain of 0.71 to 1.28 QALYs, based on HSUs derived from TTO and SG, assuming a 5% annual discounting rate (or 2.17 to 3.88 undiscounted QALYs) [32]. If left untreated, the natural history of FA is characterised by progressive deterioration with time [4], rendering these values an underestimate of true disease severity over the prescribed time horizon. These findings underscore the necessity for a formal cost-effectiveness analysis to be conducted into the use of topical sirolimus to assess its health gains in relation to the healthcare system’s implicit willingness-to-pay threshold.

The most profound impact of FA is its cosmetic burden, hence FA health states were pictorially depicted without accompanying vignettes to avoid overemphasis on rare associated complications. Instead, utilities are able to capture the psychosocial implications of the cosmesis of FA, including stigma and social anxiety, which cannot be standardised in vignettes due to interpersonal variability. However, the absence of descriptions on potential functional impacts of FA may have generated inflated HSUs and underestimated the incremental HSU of moderate FA compared to almost-clear FA. Consequently, the gain in QALYs derived from these utility values may only reflect the gain derived from cosmetic improvement when FA severity is lessened and are unable to capture potential holistic HRQoL gains due to a reduced functional impact. On the other hand, the result that moderate FA has comparable HSUs to monocular blindness suggests that visual representation of FA health states could have placed an overemphasis on cosmetic burden or introduced difficulty for participants to conceptualise cosmetic deficits against functional impacts of monocular blindness, such as loss of depth perception. Participants drawn from dermatology clinics are also more likely to have lived experience of conditions with a cosmetic burden than blindness, which may distort valuations.

Linear mixed-effects modelling found that VAS consistently produced lower HSUs than TTO and SG. This result was consistent with the literature, given that VAS is typically regarded as inferior to TTO and SG as it lacks a basis in the economic theory of trade-off. Theoretical explanations for the higher HSUs produced by TTO and SG are the influence of risk aversion, loss aversion and the framing effect [33]. Both TTO and SG are choice-based methods with reported high correlation of output values, supported by the lack of an overall statistical difference between TTO and SG HSU outputs in this study [33]. SG has been referred to as a ‘gold standard’ for direct method HSUs, however this argument lacks empirical support [34]. Nevertheless, the mean difference between TTO and SG HSUs was significant for binocular blindness (*p* = 0.003), as shown in Table S6c (Online Resource 2), suggesting that TTO and SG values may diverge for health states of greater severity, and closer mapping of these tools to one another may be warranted in future research. While TTO and SG are more cognitively demanding, the results of this study support their use as primary endpoints of future HSU estimation, while VAS may be retained as a supplementary measure. However, it should be noted that realism may have been an issue given the median age of participants was 44 years (IQR: 31.8–58.3) but a hypothetical 57 year time horizon was adopted for TTO and SG. Although this reflects the length of time patients spend in the FA health state, it potentially affected the accuracy of TTO and SG responses.

Indirect assessment tools to measure HSUs, such as the EQ-5D, were not employed in this study, but a review of studies using both direct and indirect methods found that indirect methods tend to yield lower HSUs than direct methods [14]. The EQ-5D-5L is a widely implemented indirect assessment tool for skin conditions, however its low content validity and high ceiling effect are recognised limitations [35]. While such tools may be able to factor in dimensions, such as pain, which are beyond the scope of this study, questionnaire items within other domains of mobility, self-care and usual activities are unlikely to detect significant deficits in QoL. The addition of “bolt-ons”, which are supplementary questionnaire items, to the EQ-5D-5L, such as skin irritation and self-confidence can reduce ceiling effects and would be applicable to FA [36]. Alternatively, to improve sensitivity of QoL measurement, the severity of FA could be assessed using a disease-specific QoL instrument. Similar instruments have already been developed for other conditions in dermatology, such as autoimmune bullous disease [37] and vitiligo [38]. However, these disease-specific QoL instruments are unable to measure utility values for economic appraisal. Future studies could determine HSUs for FA using indirect methods to assess accuracy of findings and highlight any disparities between health utility instruments.

The current study has calculated the HSUs for two FA health states and demonstrated significant differences between almost-clear FA and moderate FA. Minimally important difference thresholds are often used to assess clinical significance of HSUs derived from preference-based utility instruments. Although specific thresholds have not been defined for direct utility elicitation tools, the observed differences between almost-clear and moderate FA HSUs exceed published thresholds of 0.03–0.07 [39], which implies that reducing severity of FA may deliver a clinically meaningful benefit. These findings can be integrated into future studies on treatment efficacy and costing data for cost-effectiveness modelling. Formal determination of QALYs gained and healthcare cost/QALY within the context of a specific healthcare system offers critical data for policymakers to evaluate treatment of FA against competing healthcare needs. Furthermore, HSU data from this study can act as a reference point for HSU valuations across different population groups, including patient-derived utilities.

### Limitations

The findings of this study should be interpreted in the context of its limitations. Firstly, the degree of uncertainty around HSU outputs and the overlap of 95% confidence intervals should be acknowledged. This may be attributed to the large variability of participant responses within a small sample size. The sample size in this study was consistent with previous published health utility data in TSC [29], however the lack of a formal power calculation and smaller sample size compared to existing studies with comparable methodology [20, 21], may have limited the precision of effect estimates. The fixed sequence of health state presentation subjected the results to anchoring bias as participants’ response to FA health states may have been influenced by earlier blindness health states. Evaluation of accuracy is hindered by a lack of existing studies evaluating HSUs in FA.

The sociodemographics of participants were slightly skewed towards older age and higher education status and there was a greater representation of females and those of Asian ethnicity compared to the general Australian population. Although FA affect children and adolescents, only adult participants were recruited due to the nature of the HSU elicitation tasks, associated with significant cognitive demand and potential emotional distress in evaluating health states involving death. However, the most significant limitation of participant characteristics was that all participants were patients of dermatology clinics. Such participants may be more cognisant of cutaneous changes and more inclined to seek treatment for aesthetic purposes, affecting their opinion of FA health states. These factors may limit external validity and HSU comparisons between diseases.

HSUs derived from naïve participants may not reflect patient-derived utilities of FA and as previously discussed, a still image cannot convey the lived experience of FA. This may undervalue the impact of FA on QoL, limiting comparability with vignette-based HSU studies. As vignettes were not generated, members of the public with lived experience with FA were not involved in the design of this study. In addition, the FA health states were not standardised, as they do not depict the same patient, and there is a level of subjectivity associated with the assessment of severity under the Investigator’s Global Assessment score. The difference in gender between the patients shown in the two health states may also bias participant responses as assigned HSUs may have been influenced by participants’ perception of the portrayed individual, rather than solely the FA severity. Future HSU studies may benefit from standardised images, whether from the same individual or digitally generated.

A LMM was fitted for statistical analysis, despite a slight skew in variance and the bounded nature of HSU values. Alternative statistical models, such as censored normal or beta regression modelling, may have better accommodated the data, however effect estimates would be less intuitive to interpret and difficult to transform into QALYs for future economic evaluation.

## Conclusion

Overall, this study found that the perceived HSU of moderate FA was poorer than almost-clear FA across three direct measures of VAS, TTO and SG. These findings suggest that treatment to lessen the severity of FA may have a meaningful impact on QoL for patients.

## Supplementary Information

Below is the link to the electronic supplementary material.

Supplementary Material 1

Supplementary Material 2

Supplementary Material 3

## Data Availability

Data available on request from the authors.
