# Supplementary material for: Dermatology patient-derived health utility of facial angiofibroma associated with tuberous sclerosis complex
Source: Qual Life Res. 2026 Jul 30;35(9):253. doi: 10.1007/s11136-026-04362-1 (PMC13424800; doi:10.1007/s11136-026-04362-1)
Supplement: Supplementary file 1 — Supplementary Material 1 [file 11136_2026_4362_MOESM1_ESM.docx]

***Online Resource 3***

***Supplementary Statistical Methods***

The multivariable linear mixed-effects model (LMM) accounted for repeated HSUs within participants across multiple health states and elicitation tools. The fixed effects included were any demographic variables which demonstrated consistent evidence of a bivariate association in the simple linear regression (SLR), health state, assessment tool and an interaction term between health state and assessment tool.

The model can be expressed in Wilkinson and Rogers notation as:

$$HSU \sim Education+Health State*Tool+\left( 1 \right| Participant)$$

All analyses were conducted in R (version 4.5.0) using the **lme4** package and the LMM was fitted using the **lmer()** function.

Collinearity was assessed using Generalised Variance Inflation Factors (GVIFs) which were adjusted using the formula GVIF^(1/(2*df)) where (df) is the degrees of freedom for the fixed effect. A threshold of adjusted GVIF >2 was used to assess for collinearity.
