# Supplementary material for: Dermatology patient-derived health utility of facial angiofibroma associated with tuberous sclerosis complex
Source: Qual Life Res. 2026 Jul 30;35(9):253. doi: 10.1007/s11136-026-04362-1 (PMC13424800; doi:10.1007/s11136-026-04362-1)
Supplement: Supplementary file 2 — Supplementary Material 2 [file 11136_2026_4362_MOESM2_ESM.docx]

## ***Online Resource 2***

## Supplementary Tables

Table S1: Univariate linear regression analysis of sociodemographic variables associated with health state utilities for almost-clear and moderate facial angiofibroma. p-values obtained from F-statistic (bolded p-values indicate significance at α = 0.05). VAS = Visual Analogue Scale, TTO = Time Trade-Off, SG = Standard Gamble, CI = confidence interval, REF = reference category, b = regression coefficient

|  | Almost-Clear Facial Angiofibroma | | | | | | | Moderate Facial Angiofibroma | | | | | |
| --- | --- | --- | --- | --- | --- | --- | --- | --- | --- | --- | --- | --- | --- |
|  | VAS | | TTO | | SG | | | VAS | | TTO | | SG | |
|  | b | p | b | p | b | | p | b | p | b | p | b | p |
|  | [95% CI] | | [95% CI] | | [95% CI] | | | [95% CI] | | [95% CI] | | [95% CI] | |
| Age | -0.0004 | 0.707 | -0.0003 | 0.669 | 0.0001 | | 0.834 | -0.002 | 0.055 | -0.0007 | 0.395 | -0.0001 | 0.889 |
|  | [-0.002, 0.002] | | [-0.001, 0.001] | | [-0.001, 0.001] | | | [-0.005, -0.00006] | | [-0.002, 0.0009] | | [-0.001, 0.001] | |
| Sex |  |  |  |  |  | |  |  |  |  |  |  |  |
| Female | REF | 0.537 | REF | 0.076 | REF | | 0.835 | REF | 0.252 | REF | 0.053 | REF | 0.785 |
| Male | -0.020 |  | -0.035 |  | -0.004 | |  | -0.049 |  | -0.048 |  | -0.006 | |
|  | [-0.084, 0.044] | | [-0.074, 0.004] | | [-0.047, 0.038] | | | [-0.132, 0.035] | | [-0.097, 0.001] | | [-0.051, 0.039] | |
| Ethnic Group |  |  |  |  |  | |  |  |  |  |  |  |  |
| White | REF | **0.030** | REF | 0.082 | REF | | 0.736 |  | 0.464 |  | 0.328 | REF | 0.241 |
| Non-White | -0.069 |  | -0.034 |  | -0.007 | |  | -0.031 |  | -0.025 |  | -0.027 | |
|  | [-0.132, -0.007] | | [-0.073, 0.004] | | [-0.050, 0.035] | | | [-0.116, 0.053] | | [-0.075, 0.025] | | [-0.072, 0.018] | |
| Household Income (AUD) |  |  |  |  |  | |  |  |  |  |  |  |  |
| <50 200 | REF | 0.445 | REF | 0.339 | REF | | 0.269 | REF | 0.187 | REF | 0.144 | REF | 0.266 |
|  |  |  |  |  |  | |  |  |  |  |  |  |  |
| 50200-116000 | 0.061 |  | 0.003 |  | 0.051 | |  | -0.003 |  | -0.031 |  | 0.057 | |
|  | [-0.035, 0.159] | | [-0.059, 0.064] | | [-0.012, 0.114] | | | [-0.140, 0.133] | | [-0.113, 0.050] | | [-0.014, 0.128] | |
| >116000 | 0.033 |  | 0.031 |  | 0.028 | |  | 0.079 |  | 0.028 |  | 0.045 | |
|  | [-0.054, 0.121] | | [-0.025, 0.087] | | [-0.029, 0.085] | | | [-0.045, 0.203] | | [-0.046, 0.102] | | [-0.019, 0.110] | |
| Education |  |  |  |  |  |  | |  |  |  |  |  |  |
| Some high school | REF | 0.611 | REF | **0.007** | REF | **<0.001** | | REF | 0.719 | REF | 0.082 | REF | **0.026** |
| High school diploma | -0.031 |  | -0.119 |  | -0.151 |  | | -0.001 |  | -0.127 |  | -0.012 |  |
|  | [-0.187, 0.125] | | [-0.211, -0.027] | | [-0.247, -0.053] | | | [-0.207, 0.205] | | [-0.246, -0.007] | | [-0.223, -0.010] | |
| Tertiary education | -0.058 |  | -0.035 |  | -0.044 |  | | -0.045 |  | -0.060 |  | -0.033 | |
|  | [-0.192, 0.075] | | [-0.114, 0.044] | | [-0.127, 0.039] | | | [-0.221, 0.132] | | [-0.162, 0.043] | | [-0.124, 0.059] | |
| Relationship Status |  |  |  |  |  |  | |  |  |  |  |  |  |
| Committed relationship | REF | 0.994 | REF | 0.220 | REF | 0.192 | |  | 0.257 |  | 0.057 | REF | 0.208 |
| Not committed relationship | 0.0003 |  | -0.025 |  | -0.029 |  | | -0.050 |  | -0.050 |  | -0.030 | |
|  | [-0.066, 0.067] | | [-0.066, 0.015] | | [-0.073, 0.015] | | | [-0.137, 0.037] | | [-0.101, 0.002] | | [-0.076, 0.017] | |

Table S2: Fitted multivariable linear mixed model analysis-of-variance table. DF = degrees of freedom, GVIF = generalised variance inflation factor

| **Fixed Effect** | **Num DF** | **Den DF** | **F** | **p** | **Adjusted GVIF** |
| --- | --- | --- | --- | --- | --- |
| Level of Education | 2 | 96 | 1.455 | 0.239 | 1.000 |
| Tool | 2 | 1078 | 286.185 | **<0.001** | 1.414 |
| Health State | 3 | 1078 | 188.991 | **<0.001** | 1.732 |
| Tool: Health State | 6 | 1078 | 28.259 | **<0.001** | 1.682 |

Table S3: Results of fitted linear mixed effects modelling (bolded p-values indicate significance at α = 0.05). b = regression coefficient, CI = confidence interval, VAS = Visual Analogue Scale, TTO = Time Trade-Off, SG = Standard Gamble, FA = facial angiofibroma

| **Fixed Effects** | **b** | **95% CI** | **t** | **p** |
| --- | --- | --- | --- | --- |
| Intercept | 0.713 | 0.638 to 0.787 | 18.821 | **<0.001** |
| High school diploma | -0.066 | -0.150 to 0.018 | -1.541 | 0.127 |
| Tertiary education | -0.029 | -0.101 to 0.043 | -0.789 | 0.432 |
| TTO | 0.199 | 0.160 to 0.238 | 10.121 | **<0.001** |
| SG | 0.2225 | 0.186 to 0.263 | 11.421 | **<0.001** |
| Monocular blindness | 0.047 | 0.009 to 0.086 | 2.394 | **0.017** |
| Binocular blindness | -0.264 | -0.302 to -0.225 | -13.419 | **<0.001** |
| Almost-clear FA | 0.202 | 0.163 to 0.240 | 10.254 | **<0.001** |
| TTO:monocular blindness | -0.034 | -0.089 to 0.020 | -1.240 | 0.215 |
| SG:monocular blindness | -0.049 | -0.103 to 0.006 | -1.762 | 0.078 |
| TTO:binocular blindness | 0.134 | 0.079 to 0.188 | 4.804 | **<0.001** |
| SG:binocular blindness | 0.174 | 0.119 to 0.228 | 6.248 | **<0.001** |
| TTO:almost-clear FA | -0.134 | -0.188 to -0.079 | -4.810 | **<0.001** |
| SG:almost-clear FA | -0.164 | -0.218 to -0.109 | -5.885 | **<0.001** |

Table S4: Post-hoc comparison between health states. *Bonferroni-adjusted (bolded p-values indicate significance at α = 0.05). CI = confidence interval, FA = facial angiofibroma

| Health State Comparison | Difference | 95% CI | p* |
| --- | --- | --- | --- |
| Monocular blindness – binocular blindness | 0.181 | 0.151 to 0.211 | **<0.001** |
| Monocular blindness – almost-clear FA | -0.083 | -0.113 to -0.053 | **<0.001** |
| Monocular blindness – moderate FA | 0.019 | -0.011 to 0.049 | 0.542 |
| Binocular blindness – almost-clear FA | -0.264 | -0.294 to -0.234 | **<0.001** |
| Binocular blindness – moderate FA | -0.161 | -0.191 to -0.131 | **<0.001** |
| Almost-clear FA – moderate FA | 0.103 | 0.073 to 0.133 | **<0.001** |

Table S5: Post-hoc comparison between assessment tools. *Bonferroni-adjusted (bolded p-values indicate significance at α = 0.05). CI = confidence interval, VAS = Visual Analogue Scale, TTO = Time Trade-Off, SG = Standard Gamble

| Assessment Tool Comparison | Difference | 95% CI | p* |
| --- | --- | --- | --- |
| VAS – TTO | -0.132 | -0.159 to -0.106 | **<0.001** |
| VAS – SG | -0.143 | -0.169 to -0.116 | **<0.001** |
| TTO – SG | -0.011 | -0.037 to 0.016 | 1.000 |

Table S6a: Post-hoc comparison between visual analogue scale and time trade-off by health state. *Bonferroni-adjusted (bolded p-values indicate significance at α = 0.05). CI = confidence interval, VAS = Visual Analogue Scale, TTO = Time Trade-Off

| Health State | Difference (VAS – TTO) | 95% CI | p* |
| --- | --- | --- | --- |
| Monocular blindness | -0.164 | -0.203 to -0.126 | **<0.001** |
| Binocular blindness | -0.333 | -0.371 to -0.294 | **<0.001** |
| Almost-clear facial angiofibroma | -0.065 | -0.104 to -0.027 | **0.004** |
| Moderate facial angiofibroma | -0.199 | -0.238 to -0.160 | **<0.001** |

Table S6b: Post-hoc comparison between visual analogue scale and standard gamble by health state. *Bonferroni-adjusted (bolded p-values indicate significance at α = 0.05). CI = confidence interval, VAS = Visual Analogue Scale, SG = Standard Gamble

| Health State | Difference (VAS – SG) | 95% CI | p* |
| --- | --- | --- | --- |
| Monocular blindness | -0.176 | -0.214 to -0.137 | **<0.001** |
| Binocular blindness | -0.398 | -0.437 to -0.360 | **<0.001** |
| Almost-clear facial angiofibroma | -0.061 | -0.100 to -0.022 | **0.008** |
| Moderate facial angiofibroma | -0.225 | -0.263 to -0.186 | **<0.001** |

Table S6c: Post-hoc comparison between time trade-off and standard gamble by health state. *Bonferroni-adjusted (bolded p-values indicate significance at α = 0.05). CI = confidence interval, TTO = Time Trade-Off, SG = Standard Gamble

| Health State | Difference (TTO – SG) | 95% CI | p* |
| --- | --- | --- | --- |
| Monocular blindness | -0.011 | -0.050 to 0.028 | 1.000 |
| Binocular blindness | -0.066 | -0.104 to -0.027 | **0.003** |
| Almost-clear facial angiofibroma | 0.004 | -0.034 to 0.043 | 1.000 |
| Moderate facial angiofibroma | -0.026 | -0.064 to 0.013 | 0.776 |
