# Supplementary material for: Dermatology patient-derived health utility of facial angiofibroma associated with tuberous sclerosis complex
Source: Qual Life Res. 2026 Jul 30;35(9):253. doi: 10.1007/s11136-026-04362-1 (PMC13424800; doi:10.1007/s11136-026-04362-1)
Supplement: Supplementary file 3 — Supplementary Material 3 [file 11136_2026_4362_MOESM3_ESM.docx]

***Online Resource 1***


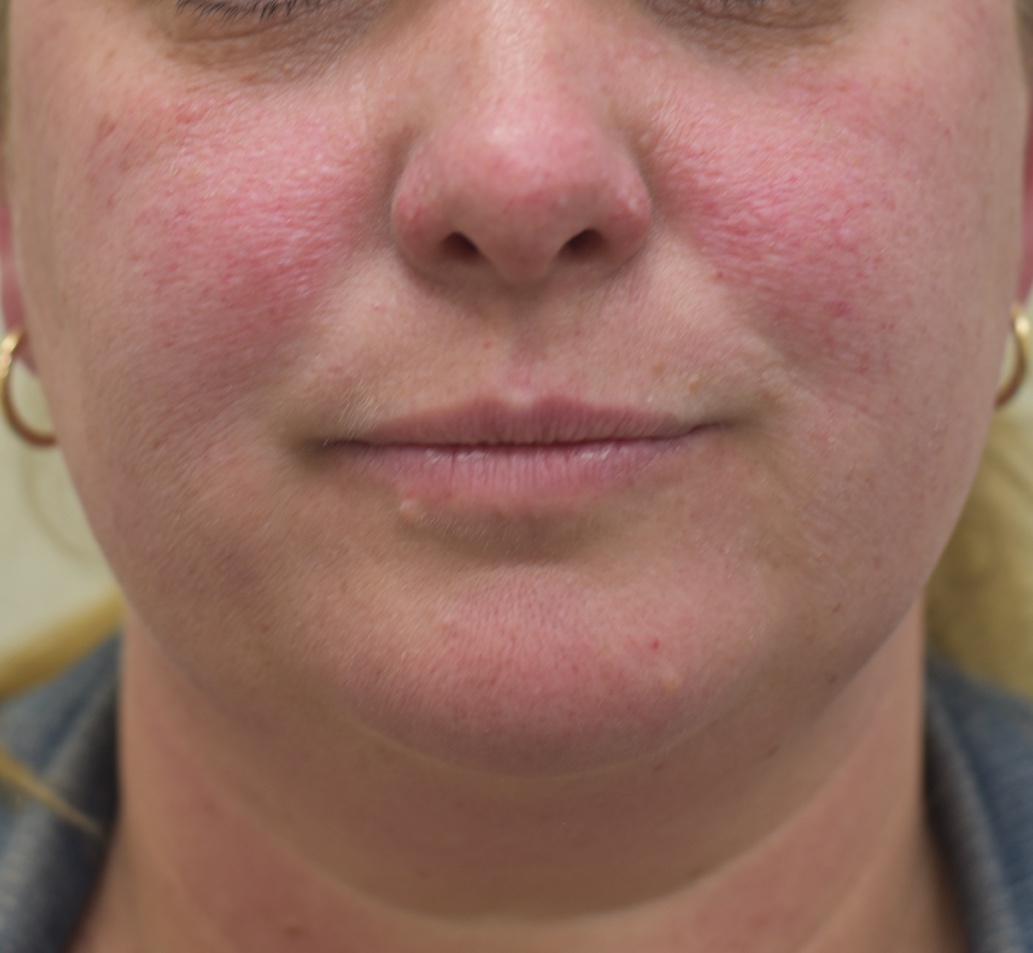


*Figure S1: Almost-clear TSC-associated FA health state*

*
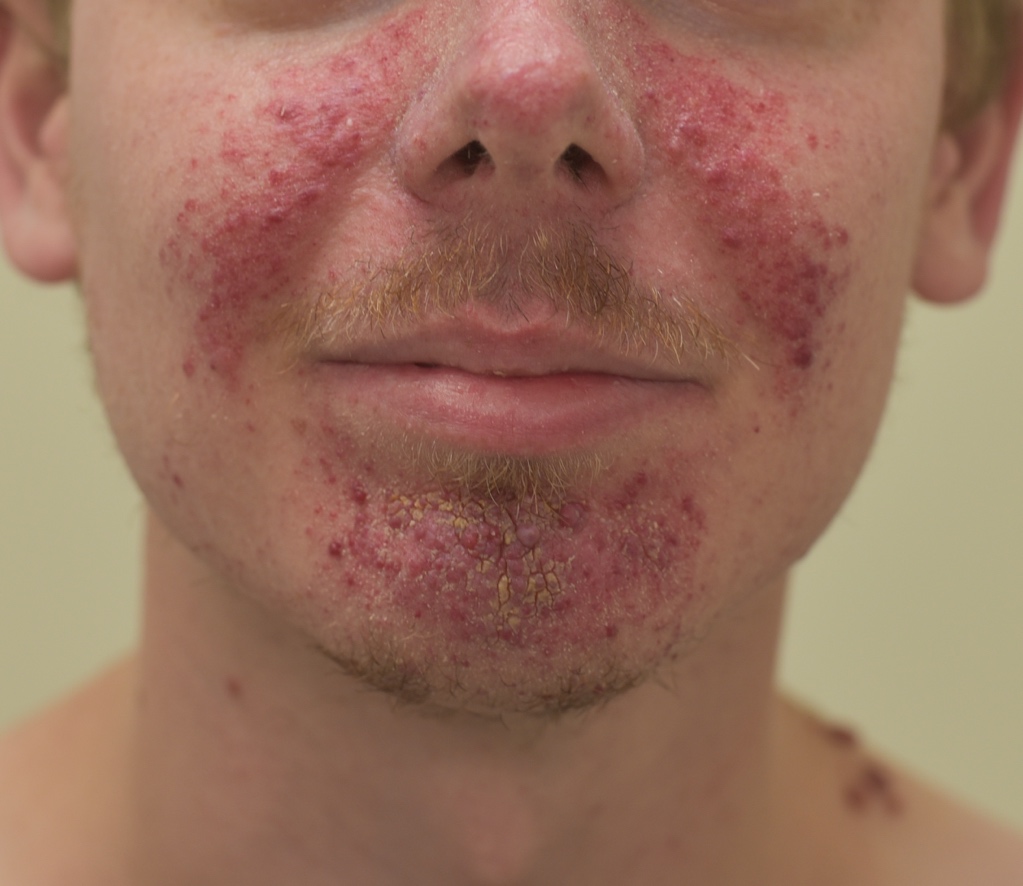
*

Figure S2: Moderate TSC-associated FA health state


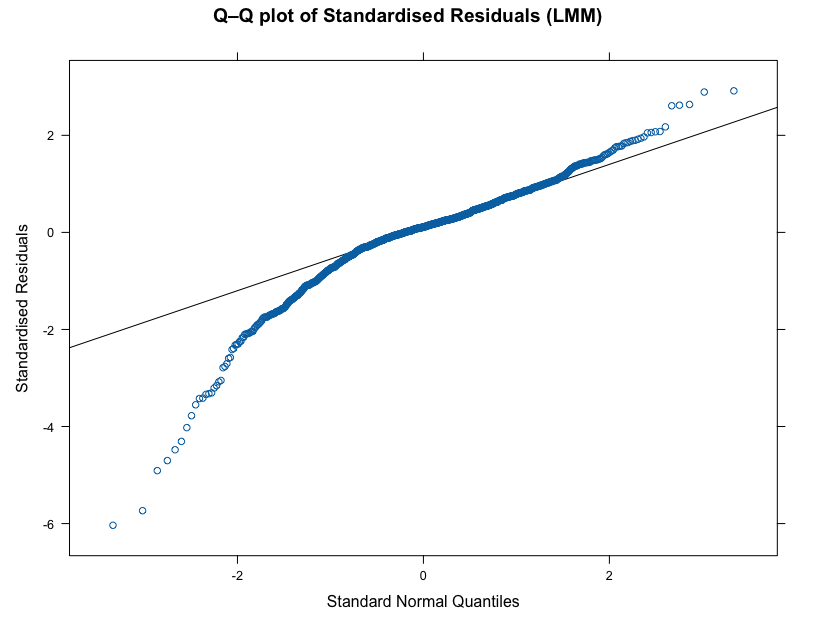


Figure S3: Q-Q plot of residuals, used for assessment of normality


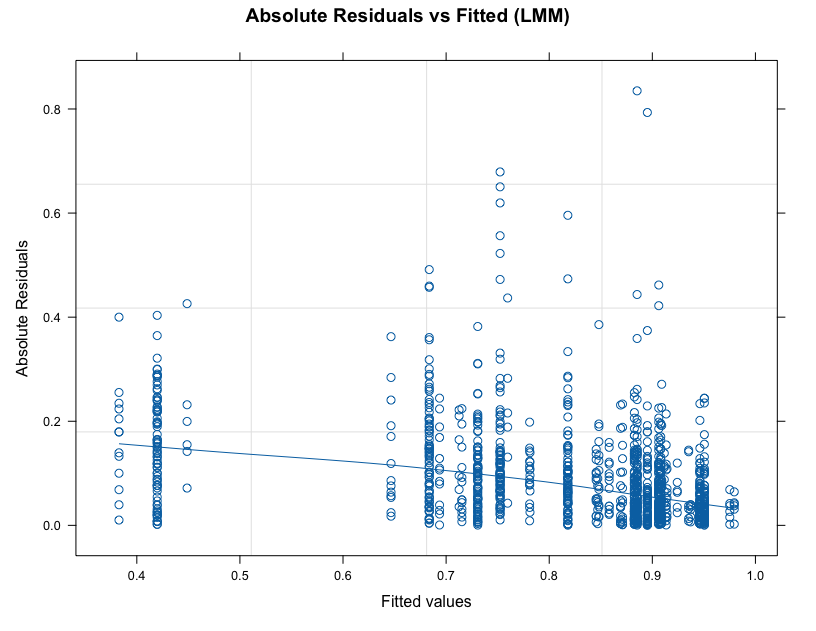


Figure S4: Absolute residuals plotted against fitted values, used for assessment of heteroscedasticity
